# Rotating hinge knee versus constrained condylar knee in revision total knee arthroplasty: A meta-analysis

**DOI:** 10.1371/journal.pone.0214279

**Published:** 2019-03-25

**Authors:** Jung-Ro Yoon, Ji-Young Cheong, Jung-Taek Im, Phil-Sun Park, Jae-Ok Park, Young-Soo Shin

**Affiliations:** 1 Department of Orthopedic Surgery, Veterans Health Service Medical Center, Seoul, South Korea; 2 Medical Library, Veterans Health Service Medical Center, Seoul, South Korea; 3 Department of Orthopedic Surgery, Chuncheon Sacred Heart Hospital, Hallym University School of Medicine, Chuncheon, South Korea; Emory University School of Medicine, UNITED STATES

## Abstract

There is debate in the literature whether rotating hinge knee (RHK) or constrained condylar knee (CCK) prostheses lead to better clinical outcomes and survival rates in patients undergoing revision total knee arthroplasty (RTKA). The purpose of this meta-analysis is to compare the survivorship and clinical outcomes of RHK and CCK prostheses. In this meta-analysis, we reviewed studies that evaluated pain and function scores, range of motion (ROM), complications, and survival rates in patients treated with RHK or CCK with short-term (<5 years) or midterm (5–10 years) follow-up. The survivorship was considered as the time to additional surgical intervention such as removal or revision of the components. A total of 12 studies (one randomized study and 11 non-randomized studies) met the inclusion criteria and were analyzed in detail. The proportion of the knees in which short-term (<5 years) survival rates (RHK, 83/95; CCK, 111/148; odds ratio [OR] 0.52; 95% CI, 0.24–1.11; P = 0.09) and midterm (5–10 years) survival rates (RHK, 104/128; CCK, 196/234; OR 1.05; 95% CI, 0.56–1.97; P = 0.88) were evaluated did not differ significantly between RHK and CCK prostheses. In addition, there were no significant differences in ROM (95% CI: -0.40 to 9.93; P = 0.07) and complication rates (95% CI: 0.66 to 2.49; P = 0.46). In contrast, CCK groups reported significantly better pain score (95% CI: 0.50 to 2.73; P = 0.005) and function score (95% CI: 0.01 to 2.00; P = 0.05) than RHK groups. This meta-analysis revealed that 87.4% of RHK and 75.0% of CCK prostheses survive at short-term (<5 years), while 81.3% of RHK and 83.8% of CCK prostheses survive at midterm (5–10 years). The differences in standardized mean pain and function scores we detected were likely to be imperceptible to patients and almost certainly below the minimum clinically important level, despite a significant difference in both groups. Based on the findings of the current meta-analysis, RHK prostheses continue to be an option in complex RTKA with reasonable midterm survivorship.

## Introduction

Revision total knee arthroplasty (RTKA) associated with bone loss and ligament instability is often more challenging than primary total knee arthroplasty with higher complication rates. [1] Thus, selecting the proper prosthesis when referring to the preoperative state of the joint is important for successful outcomes; varying levels of constraint are required in RTKA especially when dealing with bone loss and collateral insufficiency. The two commonly used prostheses of RTKA are rotating hinge knee (RHK) and constrained condylar knee (CCK). In general, RHK is a more constrained prosthesis and is considered to result in higher complication rates and lower survivorship compared to CCK; however, contemporary RHK designs have decreased constraint compared to its non-rotating predecessors, which has mitigated aseptic loosening in some studies. [2–4] The midterm results have reported on improved survivorship compared to older design without axial rotation.[1] In spite of these improvements, recurrent infection and aseptic loosening are still issues for RHK in RTKA. Therefore, CCK type implants have increased in popularity as an alternative to RHK because it can require less bone resection, has good midterm survivorship, and allows for future salvage type procedures such as RHK if necessary. [5] However, there are concerns with CCK implants; they cannot be used in all cases with high degrees of anteroposterior instability, flexion-extension gap mismatch, non-reconstructable collateral ligaments, and extensor mechanism insufficiency.[6] Although many studies have reported the clinical outcome and survivorships of patients who underwent RTKA with one of the two implants, few comparative studies exist and only one meta-analysis.[4] Published studies have not dealt with subgroup analysis regarding short-term and midterm survivorships and factors that affect survivorship of the two implants.

The primary end point of this meta-analysis was to evaluate the survival rates of short-term and midterm follow-up of RHK and CCK prostheses. The secondary end point was to compare results associated with clinical outcomes of RHK and CCK prostheses. The hypothesis is that the survival rates of short-term (<5 years) and midterm (5–10 years) follow-up are similar between RHK and CCK prostheses, but that the clinical outcomes of RHK prostheses are worse than those of CCK prostheses at final follow up.

## Materials and methods

This meta-analysis was conducted according to the guidelines of the preferred reporting items for systematic reviews and meta-analysis (PRISMA) statement (S1 PRISMA Checklist)

### Data and literature sources

This study followed the Cochrane Review Methods and the Preferred Reporting Items for Systematic Reviews and Meta-Analyses reporting guidelines for the meta-analysis of intervention trials. Although the current study involved human participants, ethical approval and informed consent from participants were not required because all data were acquired from previously published studies and analyzed anonymously without any potential harm to participants. Multiple comprehensive databases, including MEDLINE (January 1, 1976 to Feb 28, 2018), EMBASE (January 1, 1985 to Feb 28, 2018), Web of Science (January 1, 1980 to Feb 28, 2018), SCOPUS (January 1, 1980 to Feb 28, 2018), and the Cochrane Library (January 1, 1987 to Feb 28, 2018), were searched for studies that compared pain and function scores, range of motion (ROM), complications, and survival rates in patients treated with RHK or CCK with short-term (<5 years) or midterm (5–10 years) follow-up after RTKA surgery. There were no restrictions on language. Search terms used in the title, abstract, MeSH, and keywords fields were (‘knee’ [MeSH] OR ‘knee joint’ [MeSH] OR ‘knee prosthesis’ [MeSH]) AND ‘total knee arthroplasty’ [tiab] OR ‘knee prosthesis’ [tiab] OR ‘rotating hinge’ [tiab] OR ‘hinged prosthesis’ [tiab] OR ‘hinged implants’ [tiab] OR ‘RHK’ [tiab] OR ‘varus valgus’ [tiab] OR ‘VVK’ [tiab] OR ‘constrain’ [tiab] OR ‘knee’ [tiab]). After the initial online search, relevant articles and their bibliographies were manually reviewed.

### Study selection

From the title and abstract, two reviewers independently selected relevant studies for full review. The full text of the article was reviewed if the abstract did not provide enough data to make a screening decision. Studies were included in the meta-analysis if they (1) assessed human knees that had undergone RTKA utilizing RHK or CCK prosthesis; (2) had an evidence level of 1 to 3; (3) reported retrospective or prospective comparisons of surgical outcomes between groups with either RHK or CCK prosthesis in studies published after 2000, in order to avoid out-of-date prostheses models; (4) included basic data on at least one of the following five parameters: postoperative pain and function scores, ROM, complications, and survival rates. Postoperative scores on knee outcome scales included the visual analog scale (VAS), Western Ontario McMaster Universities Arthritis Index (WOMAC), American Knee Society Score (AKSS), and Knee Society Clinical Score (KSCS). A postoperative complication was defined as an adverse event of treatment recorded by the author of the study; (5) reported the number of subjects in each group (RHK and CCK prosthesis) and the means and standard deviations for the five parameters; and (6) used adequate statistical methods to compare these parameters between groups.

### Data extraction

Two reviewers independently recorded data from each study using a predefined data extraction form and resolved any differences by discussion. Recorded variables included those associated with surgical outcomes, such as postoperative pain, functional outcome, ROM, complications, and survival rates for patients with either RHK or CCK prosthesis. Sample size and the means and standard deviations of surgical outcomes in each group were also recorded. If these variables were not included in the articles, the standardized mean difference was calculated from the p-value and sample size.

### Methodological quality assessment

Two reviewers independently assessed the methodological quality of the studies. For prospective RCTs, methodological quality was assessed with the modified Jadad scale, which assesses randomization, blinding, withdrawals and dropouts, inclusion and exclusion criteria, adverse reactions, and statistical analysis. High-quality studies have scores of 4–8, whereas low-quality studies have scores of 0–3.[7,8] For the Newcastle-Ottawa Scale, as recommended by the Cochrane Non-Randomized Studies Methods Working Group, we assessed the studies based on three criteria: selection of the study groups, comparability of the groups, and ascertainment of either the exposure or the outcome of interest for case-control and cohort studies. Studies of high quality were defined as those with scores higher than 6 points. Two reviewers resolved all differences by discussion, and their decisions were subsequently reviewed by a third investigator.

### Data synthesis and analysis

The main outcomes of the meta-analysis were the proportion of cases that developed complications, survival rates, and the weighted mean difference (WMD) in ROM; however, to increase the generalizability of our results where possible, comparable continuous outcome data, including overall functional outcome and postoperative pain, were pooled across studies with use of the method of standard mean difference (SMD) with random effects. This model calculates the error term for both within-study and interstudy variability in the meta-analysis. For all comparisons, odds ratios (ORs) and 95% confidence intervals (CIs) were calculated for binary outcomes, while SMDs or WMDs and 95% CIs were calculated for continuous outcomes. For the overall functional outcome measure, we combined comparable scores from different functional outcome tools where disability was scored on a 100-point scale; the lower the score, the greater the disability. Using the same method, we combined comparable scores of postoperative pain using a 100-point scale, where 0 indicates the worst pain imaginable and 100 indicates the absence of pain. Heterogeneity was determined by estimating the proportion of between-study inconsistencies due to actual differences between studies, rather than differences due to random error or chance. I^2^ statistics with values of 25%, 50%, and 75% were considered as low, moderate, and high heterogeneity, respectively. When statistical heterogeneity was substantial, we conducted meta-regression to identify potential sources of bias, such as the study type and reason for revision. The number and age of the study subjects were also considered. All statistical analyses were performed with RevMan version 5.3 software and Stata version 14.2 static software. The risks of bias (low, high, or unclear) were independently assessed by two investigators. Publication bias was also assessed using funnel plots and Egger’s test. Subgroup analyses based on differences in follow-up period were performed for survival rates in an attempt to explore a potential source of heterogeneity. As a result, two subgroups were created in each group: short-term (<5 years) and midterm (5–10 years) survival rates. In addition, a sensitivity analysis was performed by excluding one eligible study at a time; one study [9] with a different etiology was included. Pooling of data was feasible for ROM, complications, and survival rates.

## Results

### Study identification

**Fig 1:**
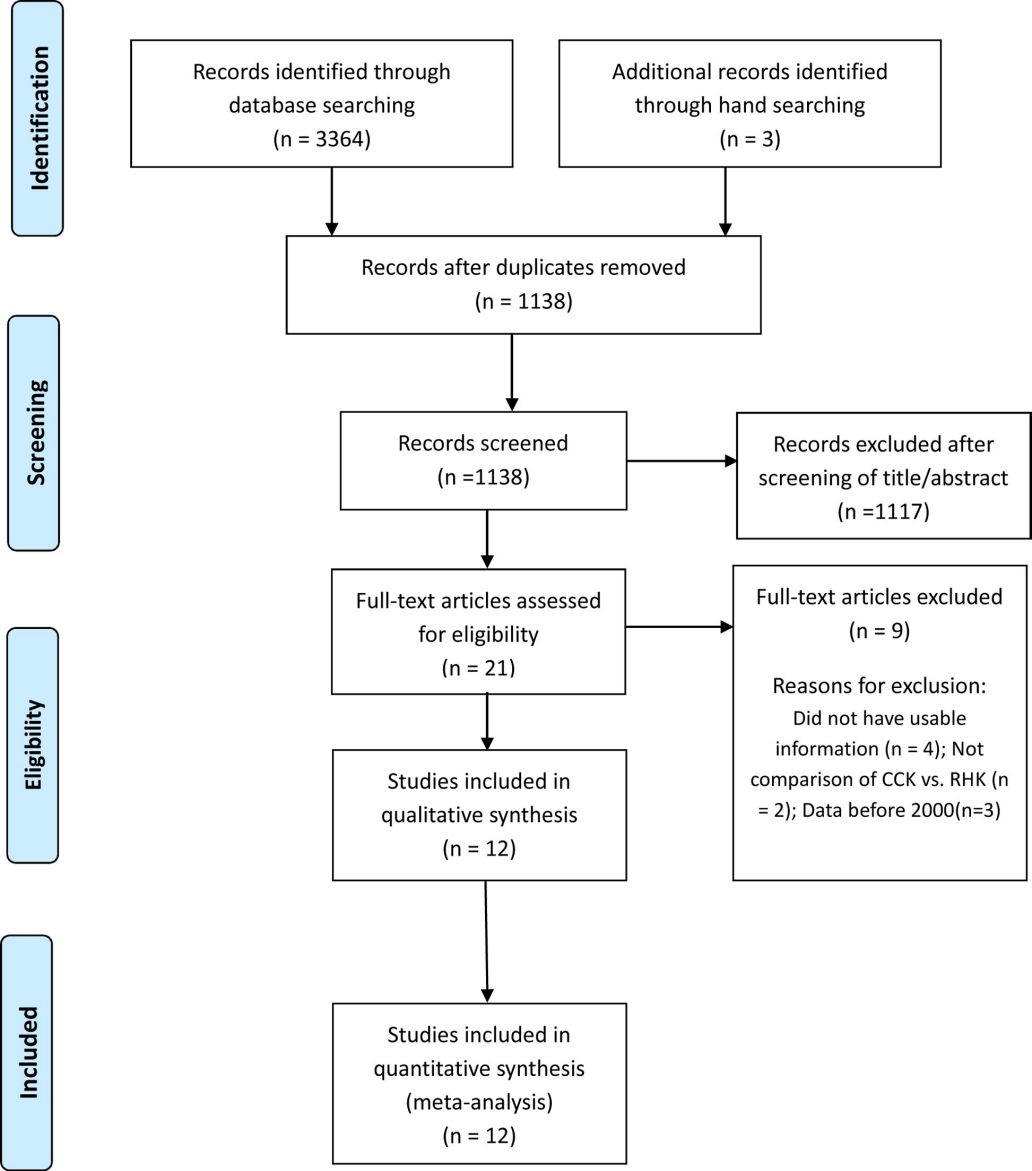
A flow diagram of preferred reporting items for systemic reviews and meta-analyses (PRISMA).

Details on study identification, inclusion, and exclusion are summarized in Fig 1. An online search yielded 583 studies in PubMed (MEDLINE), 1301 in EMBASE, 491 in Web of Science, 937 in SCOPUS, and 52 in the Cochrane Library. Four additional publications were identified through a manual search. After removing 2229 duplicate studies, 1138 studies remained. Of these studies, 1117 were excluded based on a review of the abstract or full-text article, and an additional nine studies were excluded because they had unusable information, made inappropriate group comparisons, or had data collected before 2000. This resulted in 12 studies that were included in the meta-analysis.[2, 9–19]

### Study characteristics, patient populations, quality assessment, and publication bias of the included studies

**Fig 2:**
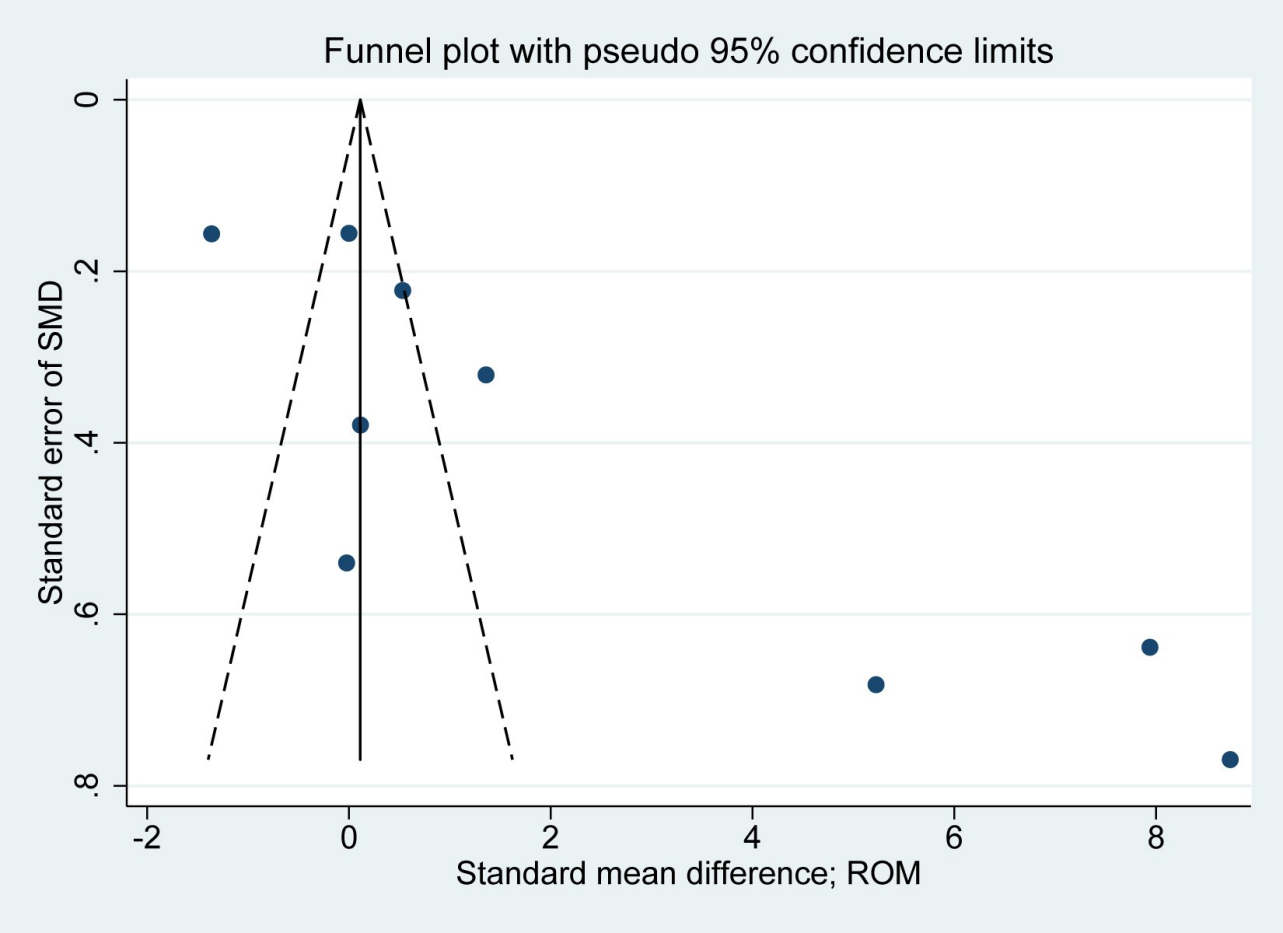
Funnel plot showing asymmetricity on range of motion (ROM).

The 12 studies we examined included 402 subjects with RHK prosthesis and 775 subjects with CCK prosthesis that had clinical outcomes reported, specific clinical scores, ROM, complications, or survival rates. Four studies (one RCT and three PCSs) compared prospectively measured parameters, whereas the other eight studies compared parameters measured by retrospective chart review. Nine studies compared groups according to ROM, seven compared survival rates, six compared pain score, five compared function score, and four compared complications (Table 1). The quality of the 12 studies included in the meta-analysis is summarized in Table 1. The one RCT was of high quality (modified Jadad scale > 4). The non-RCTs (three PCSs and eight RCSs) were of high quality (Newcastle-Ottawa Scale > 6). We only evaluated the publication bias for ROM. Funnel plot showed that the mean differences in ROM were skewed right asymmetrically, indicating some publication bias among included studies (Fig 2). Egger’s test confirmed this trend of publication bias (P = 0.007).

**Table 1:** Summary of patient characteristics of the included studies.

Study	Year	Study type	Mean age (years)		Sample size (M/F)		Prosthesis properties		Follow-up (months)		Infection/ Non-infection		Quality score	Measured parameters
			CCK	RHK	CCK	RHK	CCK	RHK	CCK	RHK	CCK	RHK		
Bali et al.[10]	2016	RCS	69.3	61.5	19(10/9)	19(16/3)	Smith, Nephew	Stryker, Biomet	Mean 48.0	Mean 36.0	10/9	10/9	NOS 8	SR
Barrack et al.[2]	2000	RCS	67.0	69.0	87(NA)	14(8/6)	Kinematic, Noiles, Insall-Burstein, LCCK	S-ROM modular	Mean 51.0	Mean 51.0	0/87	0/14	NOS 7	ROM, FS
Farfalli et al.[9]	2013	RCS	35.0	35.0	50(28/22)	36 (18/18)	Scorpio TS, Sigma PFC, LCCK	Finn knee, Lane-Burstein, Guepar	Mean 69.0	Mean 75.0	0/50	0/36	NOS 7	ROM, CR, SR
Farid et al.[11]	2013	RCS	60.7	60.0	6(2/4)	8(1/7)	Super-Stabilized Knee	Orthopedic Salvage System	Mean 34.3	Mean 32.9	0/6	0/8	NOS 7	ROM, CR, SR
Fuchs et al.[12]	2004	RCS	72.9	65.7	16(NA)	10(NA)	Genesis II	GSB	Mean 17.8	Mean 24.6	0/16	10/0	NOS 6	ROM, PS, FS
Hommel et al.[13]	2016	RCT	69.8	72.1	74 (32/42)	93 (33/69)	Legion	RT-Plus	Mean 54.0	Mean 106.0	0/74	0/93	MJS 6	PS, FS, ROM
Hossain et al.[14]	2010	PCS	65.0	65.0	149(NA)	74(NA)	Total condylar III	SMILES RMH S-ROM	Mean 57.7	Mean 57.7	NA	NA	NOS 8	PS, ROM, SR
Hwang et al.[15]	2010	RCS	65.7	65.8	15(4/11)	13(1/12)	LCCK	RHK	Mean 30.0	Mean 30.0	6/9	8/5	NOS 7	PS, FS, ROM, SR
Luttjeboer et al.[16]	2016	RCS	66.7	66.7	58 (NA)	19 (NA)	Genesis II, Legion	RT-modular, Link	Mean 24.0	Mean 24.0	0/58	0/19	NOS 9	PS, FS, ROM, CR, SR
Ritter et al.[17]	2004	RCS	NA	NA	68(NA)	4(NA)	NA	NA	Mean 28.0	Mean 28.0	NA	NA	NOS 7	ROM
Shen el al.[18]	2014	PCS	66.7	66.7	198(98/100)	94(36/58)	NA	NA	Mean 70.8	Mean 70.8	140/58	29/65	NOS 9	PS, FS, CR
Vasso et al.[19]	2013	PCS	72.0	72.0	35(NA)	18(NA)	LCCK	RHK	Mean 108.0	Mean 108.0	NA	NA	NOS 6	ROM, SR

Abbreviations: RCS, retrospective comparative study; PCS, prospective comparative study; RCT, randomized controlled trial; CCK, constrained condylar knee; RHK, rotating hinge knee; M, male; F, female; NA, not available; NOS, Newcastle-Ottawa Scale; MJS, modified Jadad scale; FS, function score; PS, pain score; CR, complication rate; SR, survival rate; ROM, range of motion

### Clinical outcomes and ROM

**Fig 3:**
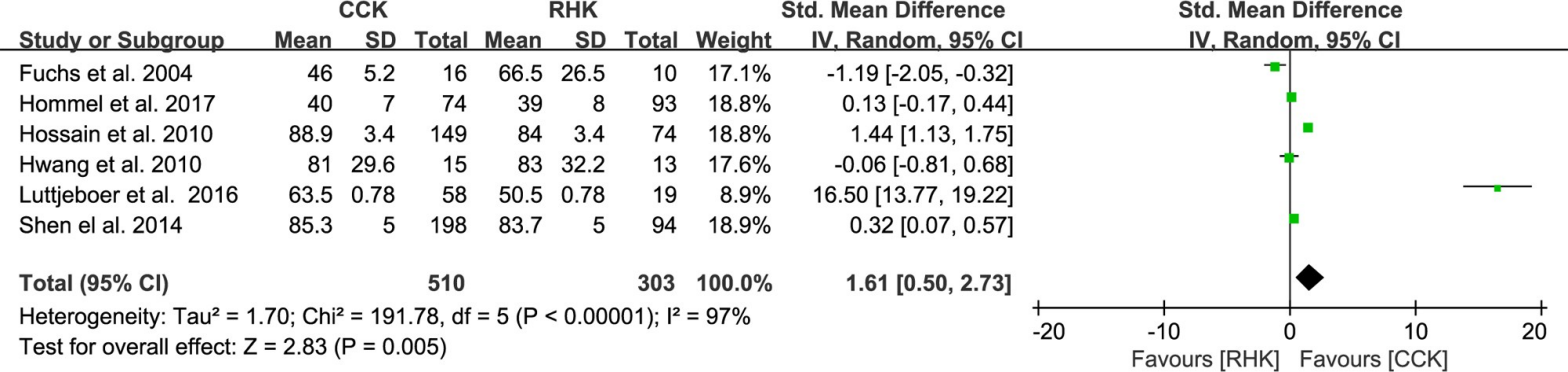
Results of aggregate analysis for comparison of pain scores between patients with constrained condylar knee (CCK) prostheses and rotating hinge knee (RHK) prostheses.

**Fig 4:**
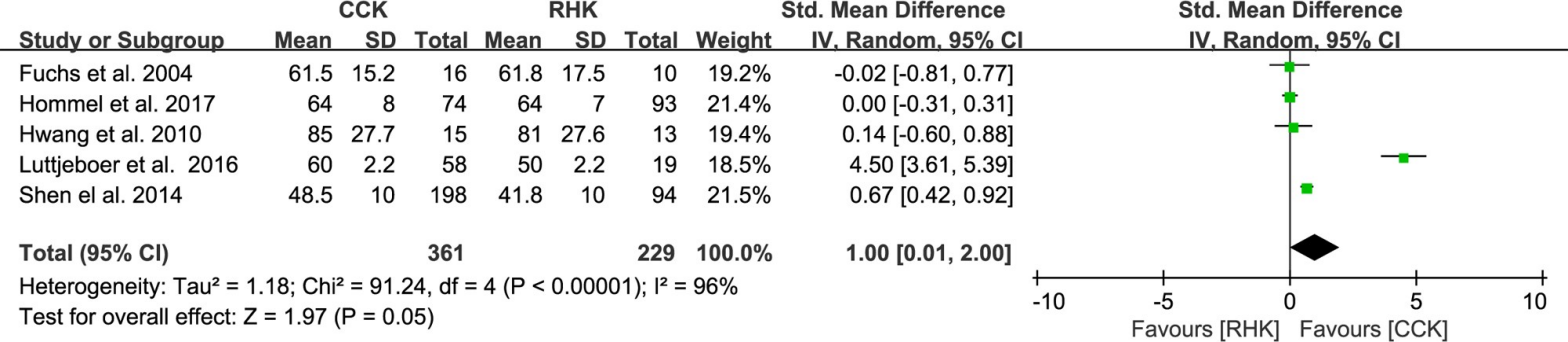
Results of aggregate analysis for comparison of function scores between patients with constrained condylar knee (CCK) prostheses and rotating hinge knee (RHK) prostheses.

**Fig 5:**
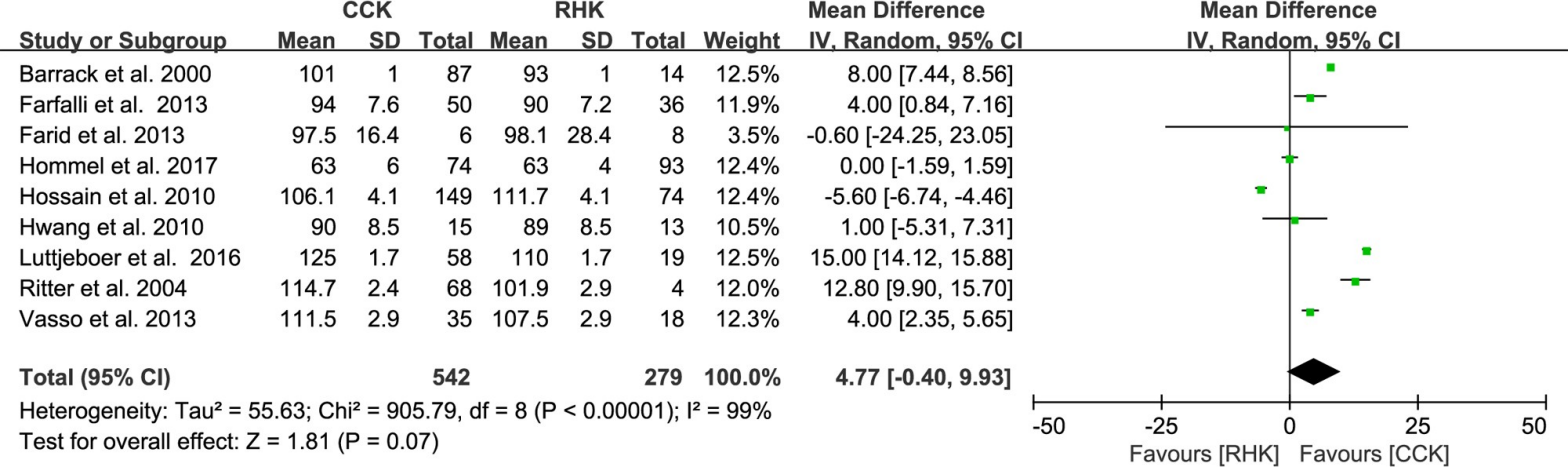
Results of aggregate analysis for comparison of postoperative range of motion (ROM) between patients with constrained condylar knee (CCK) prostheses and rotating hinge knee (RHK) prostheses.

Of the 12 studies, six compared postoperative pain between patients with RHK prosthesis (n = 303) and CCK prosthesis (n = 510). The pooled data showed that standardized mean pain score was 1.61 points higher in the CCK group than in the RHK group and was significantly different between groups (95% CI: 0.50 to 2.73 points; P = 0.005; I^2^ = 97%, Fig 3). Five studies reported postoperative function and included 229 subjects treated with RHK prosthesis and 361 subjects treated with CCK prosthesis. The pooled data showed that the standardized mean function score was 1.00 points higher in the CCK group than in the RHK group and was significantly different between groups (95% CI: 0.01 to 2.00 points; P = 0.05; I^2^ = 96%, Fig 4). Nine studies compared ROM between patients with RHK prosthesis (n = 279) and CCK prosthesis (n = 542). The pooled data showed that the mean postoperative ROM was 4.77° (95% CI: -0.40 to 9.93°; P = 0.07; I^2^ = 99%, Fig 5), with no significant difference between the RHK and CCK groups. The results of sensitivity analyses were not significantly different from those of the original analyses, indicating that the findings were robust to the decisions made in their collection process (Table 2).

**Table 2:** Sensitivity analysis.

Study	Parameter	Before exclusion	After exclusion	Statistical significance
Farfalli et al.[9] (2013)	ROM	MD = 4.77, 95% CI = -0.40 to 9.93, Z = 1.81, P = 0.07	MD = 4.86, 95% CI = -0.72 to 10.45, Z = 1.71, P = 0.09	No difference
	CR	OR = 1.28, 95% CI = 0.66,2.49, Z = 0.74, P = 0.46	OR = 1.12, 95% CI = 0.67,1.85, Z = 0.42, P = 0.67	No difference
	SR	OR = 0.77, 95% CI = 0.45,1.30, Z = 0.98, P = 0.33	OR = 0.61, 95% CI = 0.30,1.25, Z = 1.36, P = 0.18	No difference
Study	Parameter	Before exclusion	After exclusion	Statistical significance
Farfalli et al.[9] (2013)	ROM	MD = 4.77, 95% CI = -0.40 to 9.93, Z = 1.81, P = 0.07	MD = 4.86, 95% CI = -0.72 to 10.45, Z = 1.71, P = 0.09	No difference
	CR	OR = 1.28, 95% CI = 0.66,2.49, Z = 0.74, P = 0.46	OR = 1.12, 95% CI = 0.67,1.85, Z = 0.42, P = 0.67	No difference
	SR	OR = 0.77, 95% CI = 0.45,1.30, Z = 0.98, P = 0.33	OR = 0.61, 95% CI = 0.30,1.25, Z = 1.36, P = 0.18	No difference

ROM, range of motion; CR, complication rate; SR, survival rate; MD, mean difference; CI, confidence interval; OR, odd ratio

### Complications and survival rates

**Fig 6:**
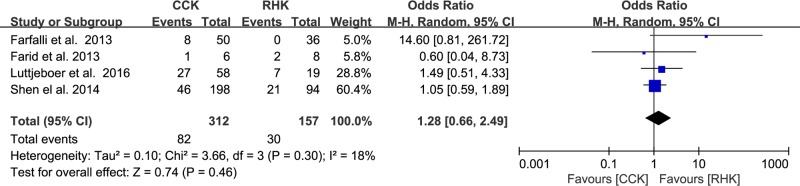
Results of aggregate analysis for comparison of complication rate between patients with constrained condylar knee (CCK) prostheses and rotating hinge knee (RHK) prostheses.

**Fig 7:**
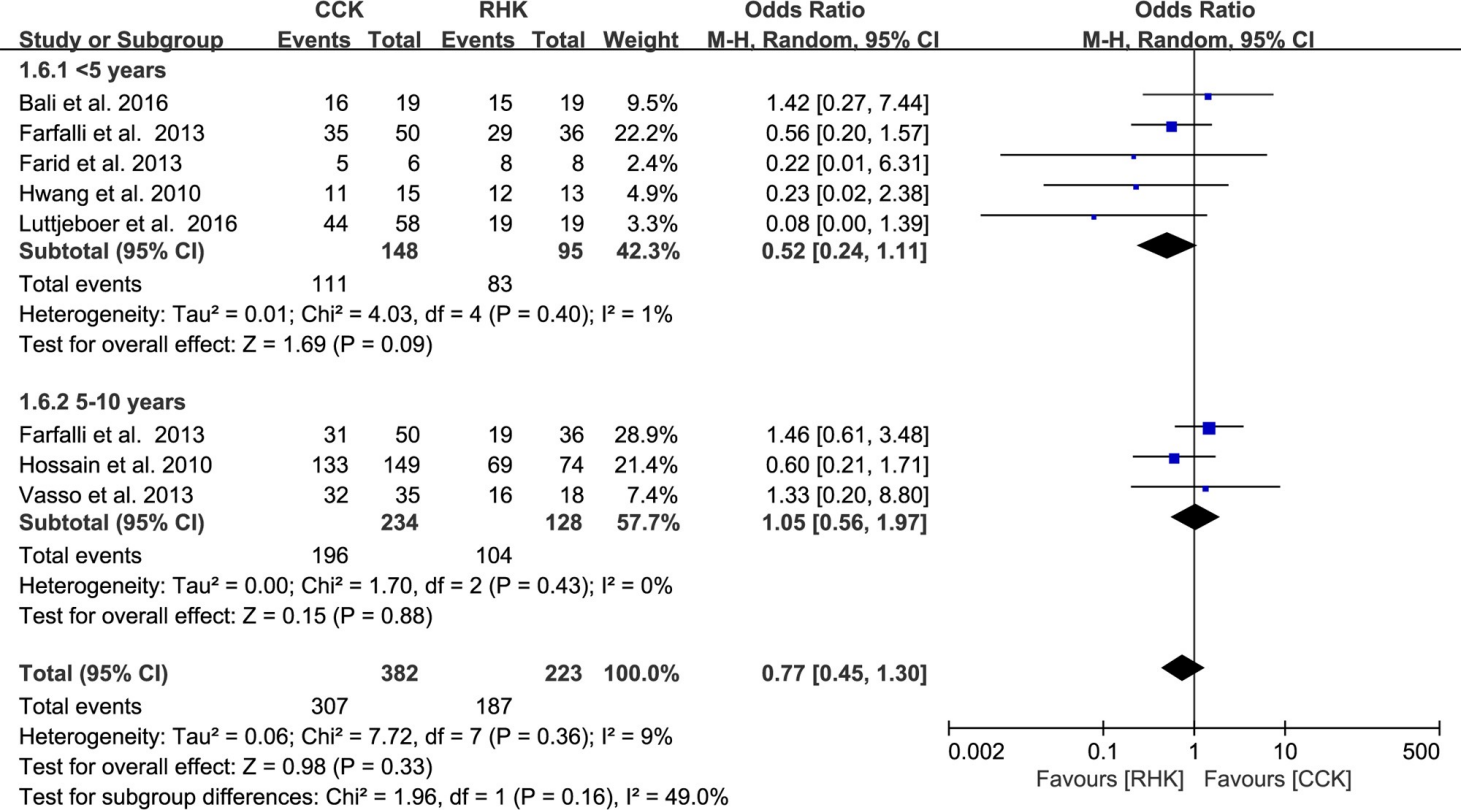
Results of aggregate analysis for comparison of short-term (<5 years) and midterm (5–10 years) survival rates between patients with constrained condylar knee (CCK) prostheses and rotating hinge knee (RHK) prostheses.

Of the 12 studies, four presented data on the proportion of subjects who developed complications, with no significant difference between groups (RHK, 30/157; CCK, 82/312; OR 1.28, 95% CI: 0.66 to 2.49; P = 0.46; I^2^ = 18%, Fig 6). Seven compared the survival rates between groups (RHK, 187/223; CCK, 307/382; OR 0.77, 95% CI: 0.45 to 1.30; P = 0.33; I^2^ = 9%, Fig 7). Five studies were assigned to the short-term (<5 years) subgroup and three studies to the midterm (5–10 years) subgroup. For the short-term (<5 years) subgroup, the RHK group had a higher survival rate than the CCK group, but this difference was not significant (RHK, 83/95; CCK, 111/148; OR 0.52, 95% CI: 0.24 to 1.11; P = 0.09; I^2^ = 1%, Fig 7). For the midterm (5–10 years) subgroup, the RHK group had a lower survival rate than the CCK group, but this difference was not significant (RHK, 104/128; CCK, 196/234; OR 1.05, 95% CI: 0.56 to 1.97; P = 0.88; I^2^ = 0%, Fig 7). The RHK group had a smaller proportion of knees with midterm survivorship (81.3%) than short-term survivorship (87.4%), whereas the CCK group had a larger proportion of knees with midterm survivorship (83.8%) than short-term survivorship (75.0%). Based on the results of sensitivity analysis, a statistical difference could not be shown compared with those of the original analysis, concluding that the findings are robust to the decisions made in their collection process (Table 2).

### Meta-regression analysis

The results of the meta-regression analysis are summarized in Table 3. For the survival rates of the RHK group, we identified the reason for revision (*P* = 0.026) as a source of heterogeneity. Similarly, we identified study type (*P* = 0.013) and reason for revision (*P* = 0.031) as sources of heterogeneity for the survival rates of CCK group. Thus, the heterogeneity in survival rates of the RHK or CCK group in the included studies was likely caused by these three factors.

**Table 3:** Meta-regression analyses of potential sources and difference in survival rate for CCK and RHK.

Variable	Coefficient	Standard error	P value	95% confidence interval
Survival rate (CCK)				
Number of patients (≤70 or ≥70)	0.091	0.084	0.331	-0.126 to 0.308
Age, mean, year (≤65 or ≥65)	0.007	0.082	0.935	-0.203 to 0.217
**Study type (RCS or Others)**	**0.140**	**0.042**	**0.020**	**0.033 to 0.246**
**Reason for revision (Infection or Others)**	**-0.148**	**0.046**	**0.024**	**-0.266 to -0.029**
Survival rate (RHK)				
Number of patients (≤70 or ≥70)	0.121	0.124	0.401	-0.273 to 0.515
Age, mean, year (≤65 or ≥65)	0.101	0.118	0.456	-0.276 to 0.478
Study type (RCS or Others)	0.131	0.097	0.267	-0.176 to 0.439
**Reason for revision (Infection or Others)**	**-0.250**	**0.060**	**0.026**	**-0.443 to -0.057**

CCK, constrained condylar knee; RHK, rotating hinge knee, RCS; retrospective comparative study

Bold value is a significant difference (P < 0.05)

## Discussion

The most important finding of this meta-analysis was that survival rates in short-term and midterm follow-ups, complication rates, and ROM did not differ significantly between patient groups with CCK or RHK prosthesis. The differences in standardized mean pain and function scores we detected were likely to be imperceptible to patients and almost certainly below the minimum clinically important level, despite a significant difference in both groups.

There is no consensus on whether CCK or RHK is superior regarding survivorship, although both prostheses are commonly used. In a recent study that compared wear of inserts between two RTKA prostheses, high-grade wear patterns such as scratching and embedded debris were more commonly seen in RHK, suggesting that RHK prostheses experience higher volumetric wear than CCK.[10] In addition, patients undergoing RHK had a tendency to have worse preoperative conditions of the knee joint because RHK is a more constrained type of prosthesis indicated for rotational instability or AORI type 3 of more severe bone defects, thereby leading to lower survival rates of RHK.[14] In contrast, RHK prostheses allow more freedom in the axial plane than CCK, even though RHK prostheses are considered to be more constrained in coronal and sagittal planes, indicating that RHK might have superior survival rates.[4] In the current meta-analysis, we found that RHK prostheses tend to have higher survival rates in short-term (<5 years) follow-up and lower in midterm (5–10 years) follow-up than those of CCK, but these differences were not significant. These findings suggest that initial stability with RHK prostheses might attribute to the superior survival rates in short-term follow-up.[15] However, this prosthesis also increased stress on the bone-cement interface, which can lead to aseptic loosening in midterm follow-up.[20] In contrast with our expectations, CCK prostheses had higher survival rates in midterm (83%) follow-up than in short-term (75%) follow-up; generally, survivorship during RTKA decreases with increasing length of follow-up. This discrepancy was likely due to groups in short-term follow-up having more patients with septic knee compared to groups in midterm follow-up. Considering the possible influence of infection as the cause of RTKA and survivorship, we further evaluated this issue by meta-regression analysis. Infection as a reason for revision appeared to be the most probable source of heterogeneity with both RHK and CCK prostheses. Indeed, more recent studies found that soft tissue integrity and poor hosts with numerous medical comorbidities of septic knee were worse after debridement and usually had higher rates of subsequent infection that require re-revision of surgery than aseptic revision.[18,21–23] Another factor that can explain these results may be the non-standardized indications and management of RTKA, suggesting that there is a bias of prosthesis choice and method of management by different surgeons, and that this is responsible for increased bone loss and poorer soft tissue integrity.[17,18,24]

Our meta-analysis also revealed that pain and functional score of RHK prostheses were significantly lower than those of CCK. Various scoring systems, including WOMAC, VAS, AKSS, and KSCS, used among 12 studies explain the results. Each of these scores was evaluated using different parameters, and KSCS is known to present a significant ceiling effect after surgery.[25] For example, preoperative KSCS of RHK was 40 points less than that of CCK due to severe deformity or instability before RTKA.[2] Another possible reason may be the RHK group having more patients with worse preoperative pain and function scores than the CCK group as a result of a severe bone defect, flexion gap imbalance, or an extensor mechanism problem caused multiple operations and infection.[15] Therefore, the postoperative clinical and function scores of RHK could be less than those of CCK, even with comparable changes in scores. Previous studies have reported greater residual pain for multiplane instability than other instabilities after surgery, indicating postoperative residual pain with RHK prostheses.[6,26] Furthermore, there were some interesting findings in a study by Shen et al. [18] that CCK prostheses had superior clinical and functional scores when used in aseptic AORI II or III bone loss, whereas patients in septic AORI II or III bone loss had better outcomes with RHK prostheses. It is possible that a more constrained prosthesis is needed in septic revision for better clinical and functional outcomes because prosthetic RHK designs allow much more aggressive capsuloligament debridement and thus more adequate infection eradication.[26]

The current meta-analysis also showed that mean postoperative ROM of CCK was 4.77 degrees higher than that of RHK, although this difference was not significant. Preoperative ROM is the most important predictive risk factor of postoperative ROM in RTKA, and patients with the highest preoperative flexion have slightly less postoperative flexion than patients with moderately higher flexion after RTKA.[27] However, we could not determine whether preoperative ROM was significantly different between the two groups owing to the limited data reported in the original papers. In addition, newer generations of RHK prostheses produced fewer patellofemoral complications and allow improved tracking because of the addition of a deeper patellofemoral groove.[28]

This study had several limitations. Firstly, as previously mentioned, a more severe preoperative state of knee joint and instability of RHK compared with those of CCK may cause selection bias. Because most studies are retrospective, preoperative clinical and function scores and preoperative ROM are lacking, which leads to limitations in reflecting preoperative differences between the two prostheses. Secondly, the heterogeneity of the included studies could also be explained by slight differences in other factors affecting surgical outcomes, such as selection of the degree of constraint, model of prosthesis, and management method of bone defects, whether using bone grafts, augmentation blocks, or metal cones. Finally, long-term results that can affect prostheses survivorship and long-term complications were not evaluated when comparing the two prostheses. In general, the 10-year survival rate of RTKA is more than 90%, and the life expectancy continues to increase.[14] Therefore, long-term follow-up is necessary for further studies.

## Conclusions

This meta-analysis revealed that 87.4% of RHK and 75.0% of CCK prostheses survive at short-term (<5 years), while 81.3% of RHK and 83.8% of CCK prostheses survive at midterm (5–10 years). The differences in standardized mean pain and function scores we detected were likely to be imperceptible to patients and almost certainly below the minimum clinically important level, despite a significant difference in both groups. Based on the findings of the current meta-analysis, RHK prostheses continue to be an option in complex RTKA with reasonable midterm survivorship.

## Supporting information

S1 PRISMA Checklist PRISMA checklist. (DOC)
