# Correction: Rotating hinge knee versus constrained condylar knee in revision total knee arthroplasty: A meta-analysis

**DOI:** 10.1371/journal.pone.0216004

**Published:** 2019-04-18

**Authors:** Jung-Ro Yoon, Ji-Young Cheong, Jung-Taek Im, Phil-Sun Park, Jae-Ok Park, Young-Soo Shin

In Table 2, the information presented was incorrectly duplicated within the table. Please view the correct Table 2 here.

**Table 2:** Sensitivity analysis.

Study	Parameter	Before exclusion	After exclusion	Statistical significance
Farfalli et al. [9] (2013)	ROM	MD = 4.77, 95% CI = -0.40 to 9.93, Z = 1.81, P = 0.07	MD = 4.86, 95% CI = -0.72 to 10.45, Z = 1.71, P = 0.09	No difference
	CR	OR = 1.28, 95% CI = 0.66,2.49, Z = 0.74, P = 0.46	OR = 1.12, 95% CI = 0.67,1.85, Z = 0.42, P = 0.67	No difference
	SR	OR = 0.77, 95% CI = 0.45,1.30, Z = 0.98, P = 0.33	OR = 0.61, 95% CI = 0.30,1.25, Z = 1.36, P = 0.18	No difference

ROM, range of motion; CR, complication rate; SR, survival rate; MD, mean difference; CI, confidence interval; OR, odd ratio
